# Comprehensive systematic review and meta-analysis of the TGF-β1 T869C gene polymorphism and autoimmune disease susceptibility

**DOI:** 10.3389/fgene.2025.1502921

**Published:** 2025-02-20

**Authors:** Yawen Zhu, Ai Qian, Yuanyuan Cheng, Ming Li, Chuanbing Huang

**Affiliations:** The First Affiliated Hospital of Anhui University of Chinese Medicine, Hefei, China

**Keywords:** autoimmune disease, meta-analysis, polymorphism, transforming growth factor-β1, susceptibility

## Abstract

**Objective:**

Autoimmune diseases (ADs) result from an aberrant immune response, in which the body mistakenly targets its own tissues. The association between TGF-β1 gene polymorphisms and risk of developing autoimmune diseases remains to be established. This meta-analysis aimed to reassess the relationship between TGF-β1 T869C gene polymorphisms and susceptibility to autoimmune diseases.

**Methods:**

We conducted a comprehensive search of seven electronic databases for case-control studies investigating the TGF-β1 T869C polymorphism in relation to autoimmune diseases, including rheumatoid arthritis, systemic lupus erythematosus, systemic sclerosis, Sjögren’s syndrome, and juvenile idiopathic arthritis. The search encompassed publications published up to June 2024. Studies were categorized by ethnicity into three groups: Asian, Caucasian, and mixed-ethnicity groups. Five different genetic models were assessed, and the quality of the included studies was evaluated using the Newcastle-Ottawa Scale (NOS). Statistical analyses were performed using Stata 14.0, by calculating the odds ratio (OR) and 95% confidence interval (CI).

**Results:**

A total of 32 case-control studies (31 articles), comprising 4,304 cases and 4,664 controls, were included in this meta-analysis. The overall analysis indicated no significant association between TGF-β1 T869C gene polymorphism and susceptibility to autoimmune diseases. However, subgroup analyses based on race and disease status revealed significant associations. Ethnic subgroup analysis showed that the TGF-β1 T869C allele model (T vs C: OR = 1.422, 95% CI = 1.109–1.824, P = 0.006), homozygous model (TT vs CC: OR = 1.923, 95% CI = 1.232–3.004, P = 0.004), and dominant model (TT + TC vs CC: OR = 1.599, 95% CI = 1.164–2.196, P = 0.004) were associated with autoimmune disease susceptibility in Asians. In the disease subgroup analysis, the results showed that the TGF-β1 T869C allele model (T vs C: OR = 1.468, 95% CI = 1.210–1.781, P = 0.000), recessive model (TT vs TC + CC: OR = 1.418, 95% CI = 1.097–1.832, P = 0.008), dominant model (TT + TC vs CC: OR = 1.747, 95% CI = 1.330–2.295, P = 0.000), homozygous model (TT vs CC: OR = 1.937, 95% CI = 1.373–2.734, P = 0.000), and heterozygous model (TC vs CC: OR = 1.555, 95% CI = 1.199–2.016, P = 0.001) were associated with rheumatoid arthritis susceptibility.

**Conclusion:**

The findings of this meta-analysis suggest that carrying the T allele of the TGF-β1 T869C polymorphism increases the risk of autoimmune diseases in Asian populations. Moreover, individuals carrying the T allele are at higher risk of developing rheumatoid arthritis.

## 1 Introduction

Autoimmune diseases (ADs) are a class of conditions characterized by immune system dysfunction leading to tissue and organ damage (Rose, 2016). These diseases are broadly categorized into organ-specific autoimmune diseases and systemic autoimmune diseases. Systemic autoimmune diseases such as rheumatoid arthritis, systemic lupus erythematosus, Sjögren’s syndrome, and systemic sclerosis are widespread and pose significant health risks (Vargas-Uricoechea, 2023). Autoimmune diseases affect approximately 10% of the global population and their prevalence is increasing (Cao et al., 2023).

The etiology of ADs is primarily attributed to a combination of genetic predispositions and environmental factors. Among the various factors involved in the pathogenesis of autoimmune diseases, cytokines have garnered considerable attention. Transforming growth factor β1 (TGF-β1) is a key cytokine predominantly expressed by immune cells such as lymphocytes, macrophages, and dendritic cells (Aoki et al., 2005). It has been reported that the TGF-β1 T869C (rs1982073) gene polymorphism is a potential risk factor for various autoimmune diseases, including rheumatoid arthritis, systemic lupus erythematosus, and systemic sclerosis (Hussein et al., 2014; Gómez-Bernal et al., 2022; Xie et al., 2022; Zhang et al., 2020). However, due to discrepancies in experimental data, some studies have found no significant association between TGF-β1 and ADs.

Before 2023, numerous meta-analyses investigated the association between the TGF-β1 promoter T869C polymorphism and the risk of autoimmune diseases. However, these studies frequently faced limitations, including small sample sizes, extended time gaps between analyses, and narrow focus on one or two autoimmune conditions. To address these challenges, the current study sought to include a broader range of autoimmune diseases and incorporate the latest literature, offering a more comprehensive evaluation of the potential connection between the TGF-β1 promoter T869C polymorphism and susceptibility to autoimmune disorders.

## 2 Materials and methods

### 2.1 Literature inclusion criteria

The studies included in this analysis were selected based on the following criteria: (i) Study Design: Only case-control studies were considered; (ii) Focus: Studies that evaluated the association between the TGF-β1 T869C polymorphism and susceptibility to autoimmune diseases, specifically rheumatoid arthritis, systemic lupus erythematosus, or systemic sclerosis; (iii) Data Requirements: Studies must provide the genotype frequency distribution for both case and control groups; (iv) Quality Control: The genotype distribution in the control group must adhere to Hardy-Weinberg Equilibrium (HWE).

### 2.2 Literature exclusion criteria

The following publications were excluded: case reports, review papers, conference abstracts, and similar non-original research articles.

### 2.3 Literature retrieval method

The meta-analysis followed the Preferred Reporting Items for Systematic Reviews and Meta-Analyses (PRISMA) guidelines. A comprehensive search was conducted to identify case-control studies that examined the association between TGF-β1 and autoimmune diseases, including rheumatoid arthritis, systemic lupus erythematosus, systemic sclerosis, Sjögren’s syndrome, and juvenile idiopathic arthritis. The databases searched included PubMed, CNKI, VIP Database, Embase, Web of Science, Wanfang Database, and SCOPUS, covering publications from the inception of each database through June 2024.

For example, the search strategy in PubMed used the following terms: ((((((((Transforming Growth Factor β1 [MeSH Terms]) OR (TGF β1 [Title/Abstract])) OR (TGF-β1 [Title/Abstract])) OR (transforming growth factor beta1 [Title/Abstract])))) AND (((((((((systemic sclerosis [MeSH Terms]) OR (scleroderma [Title/Abstract])) OR (SSC [Title/Abstract]))) OR (((rheumatoid arthritis [MeSH Terms]) OR (RA [Title/Abstract])))) OR (((systemic lupus erythematosus [MeSH Terms]) OR (SLE [Title/Abstract])))) OR (((Juvenile idiopathic arthritis [MeSH Terms]) OR (JIA [Title/Abstract])))) OR (((Sjögren syndrome [MeSH Terms]) OR (SS [Title/Abstract])))))) AND ((((((((single nucleotide [Title/Abstract])) OR (SNP [Title/Abstract])) OR (polymorphism [Title/Abstract])) OR (mutation [Title/Abstract])) OR (variation [Title/Abstract])) OR (variant [Title/Abstract])) OR (polymorphisms [Title/Abstract]))).

### 2.4 Literature extraction and quality assessment

Two researchers, Zhu Yawen and Cheng Yuanyuan, independently extracted relevant data from the articles based on the inclusion and exclusion criteria. The extracted information included the first author, year of publication, type of disease, ethnicity, and number of participants in both the case and control groups. In cases of disagreement between the two researchers, a third researcher, Qian Ai, was consulted to make a final decision. The quality of the included studies was assessed using the Newcastle-Ottawa Scale (NOS), which scores studies from 0 to 9 points. Studies with a score greater than six were considered of high quality (Mirzakhani et al., 2020).

### 2.5 Statistical analysis

Statistical analyses were performed using STATA 14.0 to assess the association between the TGF-β1 T869C gene polymorphism and autoimmune diseases. The effect size was expressed as odds ratios (OR) with 95% confidence intervals (CI). The analysis focused on five autoimmune diseases: rheumatoid arthritis, systemic lupus erythematosus, systemic sclerosis, Sjögren’s syndrome, and juvenile idiopathic arthritis.

The chi-square test was used to evaluate Hardy-Weinberg Equilibrium (HWE) in each control group. If heterogeneity among studies was indicated by a p-value of less than 0.05, a random-effects model was applied; otherwise, a fixed-effects model was used. The analysis included various genetic models: recessive (TT vs. TC + CC), dominant (TT + TC vs. CC), allele (T vs. C), homozygous (TT vs. CC), and heterozygous (TC vs. CC). Subgroup analysis was conducted when the number of studies was allowed. Publication bias was assessed using the Begg’s and Egger’s tests.

## 3 Research results

### 3.1 Literature retrieval

**FIGURE 1:**
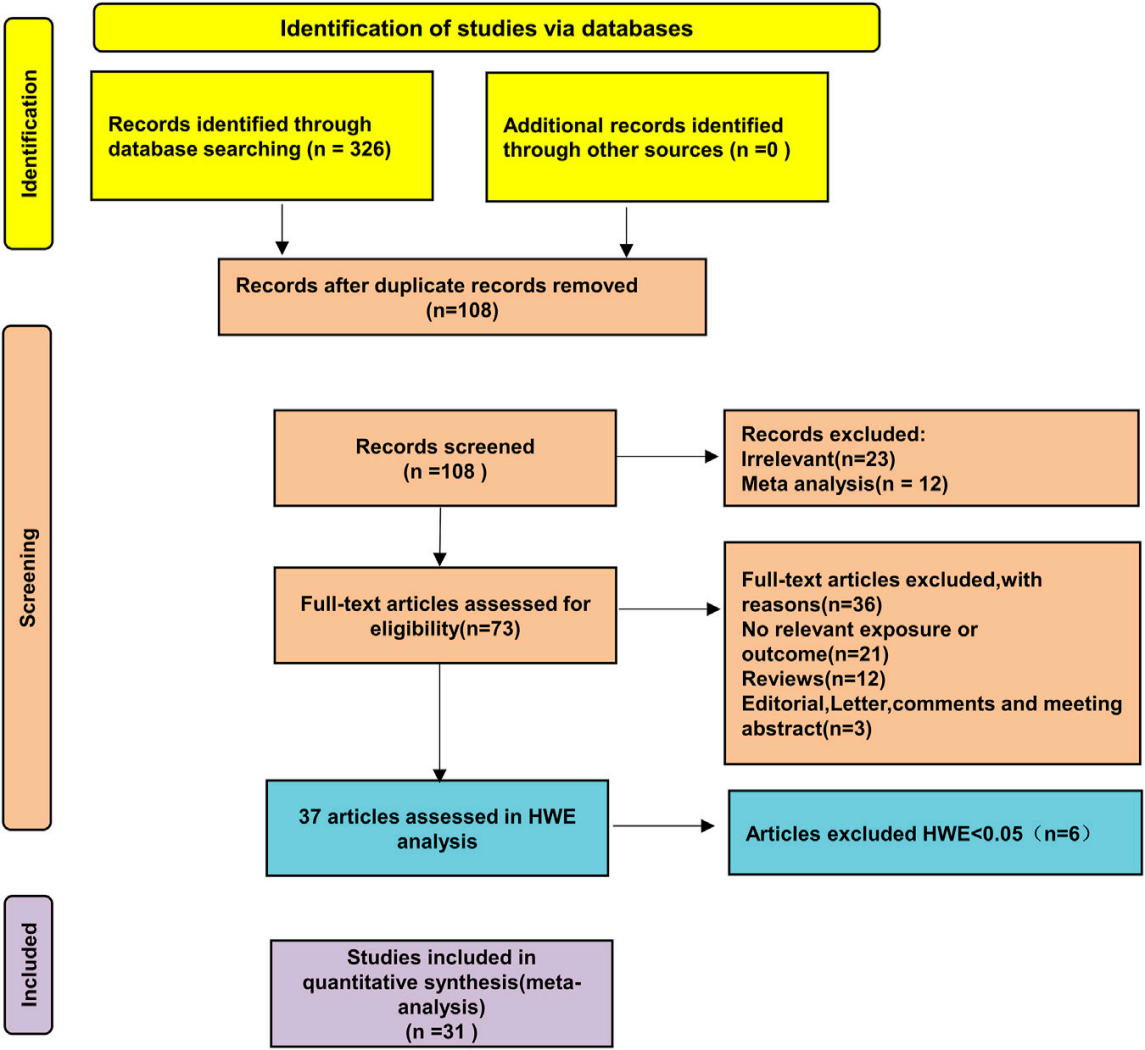
Schematic diagram of the literature screening process.

A preliminary search identified 326 published papers (Figure 1). After removing duplicates, 108 papers remained for the next screening phase. Based on a review of the titles and abstracts, 37 articles met the inclusion criteria and were selected for further analysis. After conducting Hardy-Weinberg Equilibrium (HWE) analysis, 31 articles (32 studies) were included in this meta-analysis, comprising 4,304 patients with autoimmune diseases and 4,664 healthy controls (Table 1). The studies included 14 focused on Asian populations, 13 on Caucasian populations, and 5 on mixed ethnic groups. The disease-specific studies included 7 studies on systemic sclerosis, 6 on systemic lupus erythematosus, 17 on rheumatoid arthritis, and one each on Sjögren’s syndrome and juvenile idiopathic arthritis.

**TABLE 1:** Basic information on included studies.

Number	Author	Year	Country	Racial	Disease number	Control number	Disease	Nos	Hwe
No. 1	Lomeli-Nieto et al. (2022)	2022	Mexico	Mixed	56	120	SSC	7	0.976
No. 2	Büyük et al. (2010)	2010	Turkey	Caucasian	43	75	SSC	8	0.224
No. 3	Sugiura et al. (2003)	2003	Japan	Asia	87	110	SSC	8	0.952
No. 4	Lee et al. (2004)	2003	South Korea	Asia	61	148	SSC	8	0.886
No. 5	Zhou et al. (2000)	2000	America	Mixed	19	76	SSC	8	1
No. 6	Beretta et al. (2008)	2008	Italy	Caucasian	242	242	SSC	8	1
No. 7	Ohtsuka et al. (2002)	2002	Japan	Asia	59	110	SSC	8	0.93
No. 8	Paradowska-Gorycka et al. (2019)	2019	Polish	Caucasian	216	552	SLE	8	0.68
No. 9	Wang B. et al. (2007)	2007	Japan	Asia	196	106	SLE	8	0.536
No. 10	Sayed et al. (2014)	2014	Egypt	Caucasian	56	40	SLE	7	0.998
No. 11	Stadtlober et al. (2021)	2021	Brazil	Mixed	203	165	SLE	9	0.315
No. 12	Lu et al. (2004)	2004	China	Asia	138	182	SLE	7	0.574
No. 13	Guarnizo-Zuccardi et al. (2007)	2007	Columbia	Mixed	120	102	SLE	7	0.15
No. 14	Alayli et al. (2009)	2009	Turkey	Caucasian	131	133	RA	9	0.999
No. 15	Hussein et al. (2014)	2014	Egypt	Caucasian	160	168	RA	7	0.358
No. 16	Kim et al. (2004)	2004	South Korea	Asia	143	148	RA	8	0.337
No. 17	Panoulas et al. (2009)	2009	England	Caucasian	395	401	RA	9	1
No. 18	Patel et al. (2020)	2020	North India	Asia	76	87	RA	8	0.984
No. 19	Pokorny et al. (2003)	2003	New Zeeland	Caucasian	117	140	RA	8	0.809
No. 20	Saad et al. (2015)	2015	Egypt	Caucasian	105	80	RA	8	0.246
No. 21	Shaker et al. (2016)	2016	Egypt	Caucasian	104	90	RA	8	0.232
No. 22	Sugiura et al. (2002)	2002	Japan	Asia	155	110	RA	9	0.952
No. 23	Sun et al. (2019)	2019	China	Asia	150	150	RA	8	0.81
No. 24	Hassan et al. (2022)	2022	Egypt	Caucasian	30	30	RA	8	0.47
No. 25	Wang Y. P. et al. (2007)	2007	China	Asia	105	110	RA	8	0.827
No. 26	Li (2011)	2011	China	Asia	112	201	RA	9	0.817
No. 27	Zhu et al. (2006)	2006	China	Asia	76	100	RA	9	0.908
No. 28	Iriyoda et al. (2020)	2020	Brazil	Mixed	262	168	RA	9	0.694
No. 29	Kobayashi et al. (2009)	2015	Japan	Asia	137	117	RA	8	0.811
No. 30	Kobayashi et al. (2009)	2015	Japan	Asia	137	108	RA	8	0.846
No. 31	Gottenberg et al. (2004)	2004	French	Caucasian	128	88	SS	8	0.47
No. 32	Oen et al. (2005)	2005	Canada	Caucasian	149	92	JIA	7	0.43

### 3.2 Meta-analysis of TGF-β1 T869C gene polymorphism and susceptibility to autoimmune diseases

Table 2 presents a summary of the meta-analysis findings regarding the potential association between TGF-β1 T869C gene polymorphism and susceptibility to autoimmune diseases. Because of the heterogeneity observed across all five genetic models, a random effects model was used for the analysis. The findings show no significant association between the TGF-β1 T869C gene polymorphism and susceptibility to autoimmune diseases (T vs. C: OR = 1.163, 95% CI = 1.010-1.339, P = 0.036; TT vs. CC: OR = 1.398, 95% CI = 1.074-1.820, P = 0.013; TT vs. TC + CC: OR = 1.156, 95% CI = 0.966-1.382, P = 0.113; TT + TC vs. CC: OR = 1.219, 95% CI = 0.998-1.504, P = 0.065; TC vs CC: OR = 1.151, 95% CI = 0.950-1.395, P = 0.151).

**TABLE 2:** Meta-analysis results of TGF-β1 T869C and autoimmune diseases.

rs1982073	Comparison	No.of studies	Test of association			Model	Test of heterogeneity			Publication bias	
			OR	95% CI	P Value		Q	P Value	I2 (%)	P begg	P egger
Overall	TT versus CC CT	32	1.156	0.966–1.382	0.113	R	93.32	0.000	66.8	0.593	0.265
	TT TC versus CC	32	1.219	0.988–1.504	0.065	R	112.37	0.000	72.4	0.808	0.464
	T versus C	32	1.163	1.010–1.339	0.036	R	149.82	0.000	79.3	0.277	0.187
	TT versus CC	32	1.398	1.074–1.820	0.013	R	123.41	0.000	74.9	0.178	0.214
	TC versus CC	32	1.151	0.950–1.395	0.151	R	82.81	0.000	62.6	0.987	0.767

### 3.3 Racial asian meta-analysis of TGF-β1 T869C gene polymorphism

**FIGURE 2:**
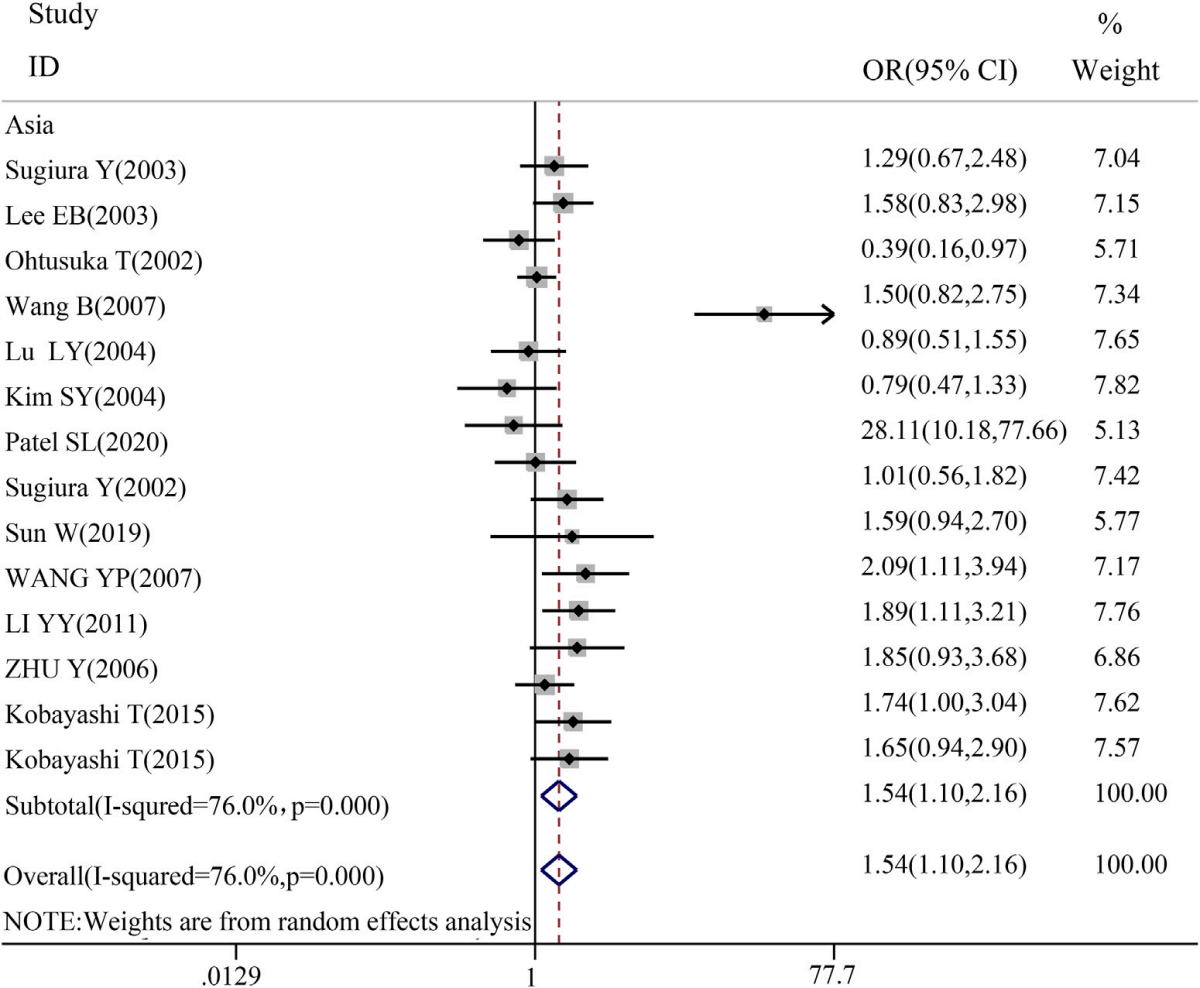
Forest map of Asian recessive gene model.

To gain a deeper understanding of the relationship between TGF-β1 T869C gene polymorphism and susceptibility to autoimmune diseases, an ethnic subgroup analysis was conducted. Quantitative analysis of 32 eligible studies identified three distinct ethnic groups: Asians (14 studies), Caucasians (13 studies), and mixed ethnic groups (5 studies). Racial analysis indicated a significant association between TGF-β1 T869C gene polymorphism and susceptibility to autoimmune diseases in the Asian population (Table 3). The results for the Asian subgroup are as follows: TT vs. TC + CC: OR = 1.540, 95% CI = 1.099–2.157, P = 0.012 < 0.01; TT + TC vs. CC: OR = 1.599, 95% CI = 1.164–2.196, P = 0.004 < 0.01; T vs. C: OR = 1.422, 95% CI = 1.109–1.824, P = 0.006 < 0.01; TT vs. CC: OR = 1.923, 95% CI = 1.232–3.004, P = 0.004 < 0.01; TC vs. CC: OR = 1.383, 95% CI = 1.074–1.782, P = 0.012 < 0.01. These findings highlight a significant association between the TGF-β1 T869C polymorphism and susceptibility to autoimmune disease in the Asian population. However, no significant association was observed between Caucasian and mixed-race populations. A forest plot for the Asian group, using the recessive genetic model as an example, is illustrated in Figure 2.

**TABLE 3:** Ethnic subgroup analysis of TGF-β1 T869C and autoimmune diseases.

rs1982073	Comparison	No.of studies	Test of association			Model	Test of heterogeneity			Publication bias	
			OR	95% CI	P Value		Q	P Value	I2 (%)	P begg	P egger
Asian	TT versus CC CT	14	1.540	1.099–2.157	0.012	R	54.06	0.000	76.0	-	-
	TT TC versus CC	14	1.599	1.164–2.196	0.004	R	49.18	0.000	73.6	-	-
	T versus C	14	1.422	1.109–1.824	0.006	R	82.74	0.000	84.3	-	-
	TT versus CC	14	1.923	1.232–3.004	0.004	R	62.09	0.000	79.1	-	-
	TC versus CC	14	1.383	1.074–1.782	0.012	R	27.48	0.011	52.7	-	-
Mixed	TT versus CC CT	5	0.947	0.732–1.224	0.676	F	4.68	0.322	14.5	-	-
	TT TC versus CC	5	0.744	0.562–0.986	0.039	F	7.37	0.117	45.8	-	-
	T versus C	5	0.888	0.756–1.043	0.146	F	7.67	0.105	47.8	-	-
	TT versus CC	5	0.892	0.400–1.992	0.781	R	19.70	0.001	79.7	-	-
	TC versus CC	5	0.747	0.557–1.003	0.052	F	5.92	0.206	32.4	-	-
Caucasian	TT versus CC CT	13	0.947	0.824–1.089	0.444	F	18.14	0.111	33.9	-	-
	TT TC versus CC	13	1.101	0.820–1.478	0.523	R	33.82	0.001	64.5	-	-
	T versus C	13	1.043	0.892–1.220	0.598	R	29.93	0.003	59.9	-	-
	TT versus CC	13	1.125	0.833–1.518	0.443	R	26.48	0.009	54.7	-	-
	TC versus CC	13	1.114	0.803–1.547	0.517	R	36.98	0.000	67.5	-	-

### 3.4 Disease-specific subgroup analysis of TGF-β1 T869C gene polymorphism

**FIGURE 3:**
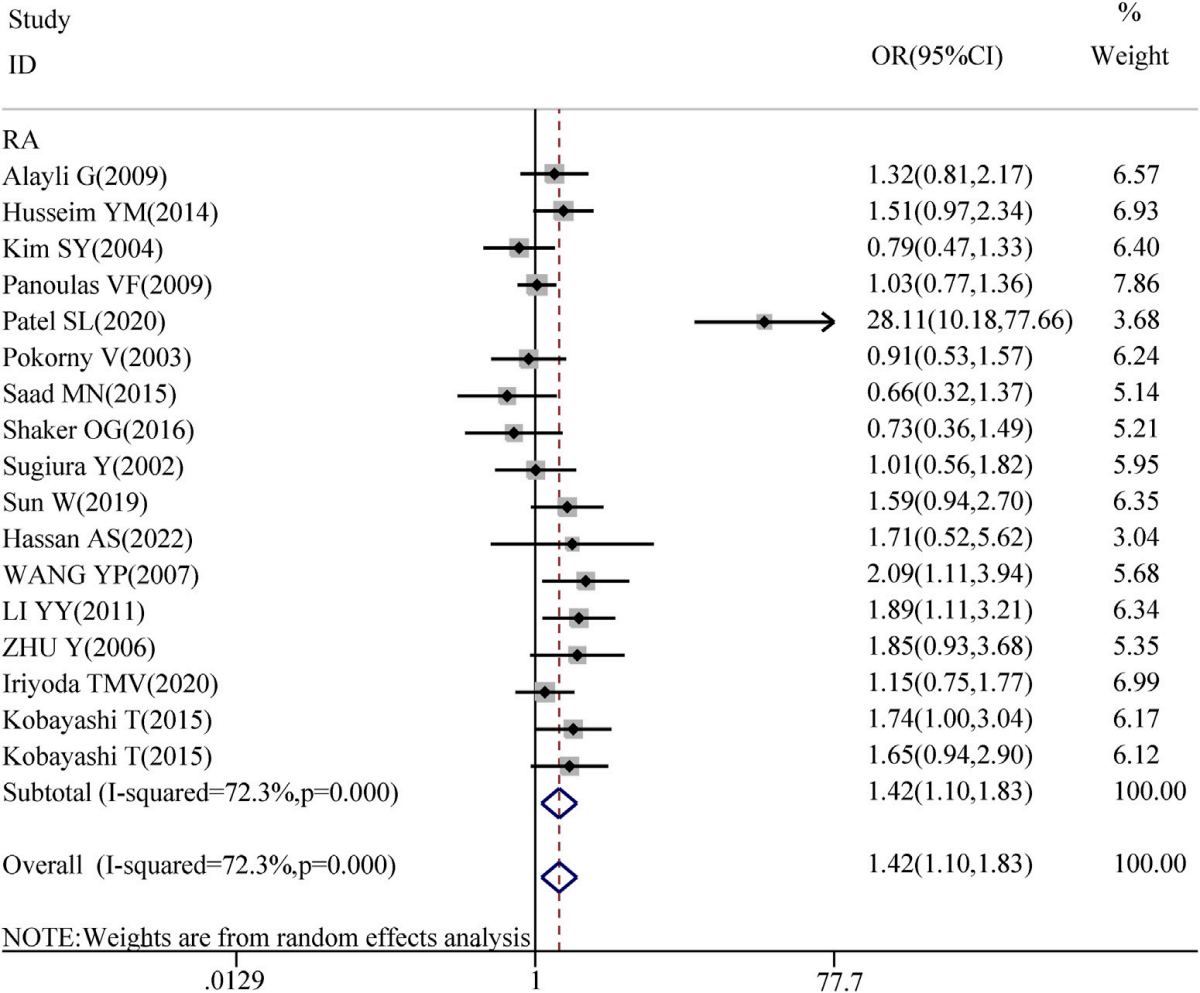
Forest map of RA recessive gene model.

The association between TGF-β1 T869C gene polymorphism and specific autoimmune diseases, including rheumatoid arthritis (RA), systemic lupus erythematosus (SLE), and systemic sclerosis (SSC), was assessed using disease-specific subgroup analysis. The analysis included 17 studies on RA, 7 on SLE, and 6 on SSC, as shown in Table 4. In the case of rheumatoid arthritis, all five genetic models revealed a significant correlation: TT vs. TC + CC: OR = 1.418, 95% CI = 1.097–1.832, P = 0.008 < 0.01; TT + TC vs. CC: OR = 1.747, 95% CI = 1.330–2.295, P = 0.000 < 0.01; T vs. C: OR = 1.468, 95% CI = 1.210–1.781, P = 0.001 < 0.01; TT vs. CC: OR = 1.937, 95% CI = 1.373–2.734, P = 0.001 < 0.01; TC vs. CC: OR = 1.555, 95% CI = 1.199–2.016, P = 0.001 < 0.01. However, no significant association was observed between TGF-β1 T869C gene polymorphism and susceptibility to SLE or SSC. A forest plot for the RA subgroup, illustrating the recessive genetic model, is shown in Figure 3.

**TABLE 4:** TGF-β1 T869C and autoimmune diseases disease grouping analysis.

Diseases	Comparison	No.of studies	Test of association			Model	Test of heterogeneity		
			OR	95% CI	P Value		Q	P	I2 (%)
SSC	TT versus CC CT	7	0.879	0.593–1.303	0.520	R	12.47	0.052	51.9
	TT TC versus CC	7	0.804	0.559–1.154	0.237	R	11.43	0.076	47.5
	T versus C	7	0.869	0.664–1.138	0.307	R	16.03	0.014	62.6
	TT versus CC	7	0.739	0.426–1.281	0.281	R	16.4	0.012	63.4
	TC versus CC	7	0.869	0.667–1.132	0.297	F	7.5	0.277	20.0
SLE	TT versus CC CT	6	0.914	0.736–1.136	0.418	F	6.41	0.268	22.1
	TT TC versus CC	6	0.900	0.715–1.132	0.366	F	5.61	0.346	10.9
	T versus C	6	0.932	0.816–1.066	0.304	F	5.93	0.313	15.7
	TT versus CC	6	1.479	0.895–2.444	0.127	R	15.74	0.008	68.2
	TC versus CC	6	0.914	0.719–1.162	0.464	F	5.81	0.326	13.9
RA	TT versus CC CT	17	1.418	1.097–1.832	0.008	R	57.82	0	72.3
	TT TC versus CC	17	1.747	1.330–2.295	0.000	R	54.33	0	70.5
	T versus C	17	1.468	1.210–1.781	0.000	R	82.24	0	80.5
	TT versus CC	17	1.937	1.373–2.734	0.000	R	61.14	0	73.8
	TC versus CC	17	1.555	1.199–2.016	0.001	R	43.26	0	63.0
SS	TT versus CC CT	1	1.104	0.608–2.006	0.745	-	-	-	-
	TT TC versus CC	1	0.676	0.333–1.372	0.278	-	-	-	-
	T versus C	1	0.910	0.618–1.338	0.631	-	-	-	-
	TT versus CC	1	0.780	0.345–1.762	0.550	-	-	-	-
	TC versus CC	1	0.622	0.296–1.309	0.211	-	-	-	-
JIA	TT versus CC CT	1	0.570	0.323–1.005	0.052	-	-	-	-
	TT TC versus CC	1	0.356	0.168–0.756	0.007	-	-	-	-
	T versus C	1	0.584	0.401–0.850	0.005	-	-	-	-
	TT versus CC	1	0.287	0.124–0.666	0.004	-	-	-	-
	TC versus CC	1	0.403	0.184–0.882	0.023	-	-	-	-

### 3.5 Sensitivity analysis and publication bias assessment

**FIGURE 4:**
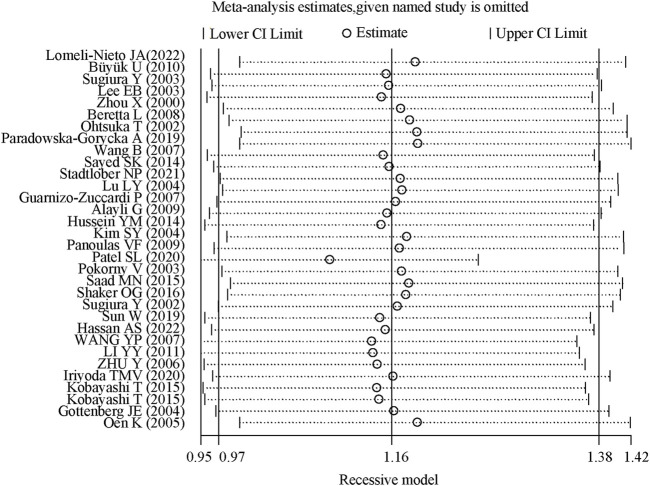
Sensitivity analysis of TGF-β1 T869C polymorphism.

The sensitivity analysis demonstrated that the odds ratios (OR) and 95% confidence intervals (CI) for the comparisons TT + TC vs. CC, TT vs. TC + CC, T vs. C, TT vs. CC, and TC vs. CC remained consistent. This indicates that the results were statistically robust, as illustrated in Figure 4.

**FIGURE 5:**
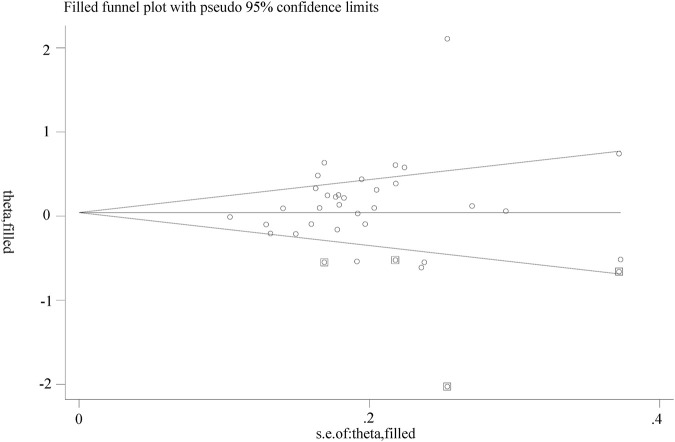
Begg funnel plot.

A funnel plot that showed no apparent asymmetry was constructed to assess publication bias. Additionally, both Begg’s test and Egger’s test were performed, and neither test provided evidence of publication bias (T vs. C: Begg’s test, P = 0.277; Egger’s test, P = 0.187), as shown in Figure 5.

## 4 Discussions

Autoimmune diseases are characterized by the immune system erroneously recognizing normal tissues and cells as foreign entities, leading to an aggressive immune response. Although the precise mechanisms underlying these conditions remain elusive, they are thought to result from a complex interplay between genetic predisposition, environmental influences, gender differences, and epigenetic modifications (Li et al., 2020; Kwiatkowska and Maślińska et al., 2020). Cytokines, a diverse group of small proteins, are central in driving autoimmune responses. Cytokines play a vital role in regulating and mediating immune system functions, and their signaling is essential for a range of biological processes, including cell development, tissue repair, aging, and immune responses (de Gruijter et al., 2022; Lau et al., 2021).

Transforming Growth Factor Beta (TGF-β) is a multifunctional cytokine belonging to a family of growth factors present in the extracellular matrix. Its primary function is to suppress immune cell activity and prevent excessive immune response (Li et al., 2006). The TGF-β family consists of several subtypes including TGF-β1, TGF-β2, and TGF-β3. Among these, TGF-β1 plays a crucial role in the development of autoimmune diseases by inhibiting the activity of T cells, B cells, macrophages, and other immune cells (Prud’homme and Piccirillo et al., 2000). Polymorphisms in TGF-β1 may theoretically affect its expression, potentially increasing the risk of autoimmune diseases. Research has indicated that TGF-β1 produced by regulatory T cells (Tregs) is essential for modulating allergic and autoimmune responses. However, a gradual reduction in TGF-β1 transcription within Tregs leads to a pronounced immune dysregulation (Turner et al., 2020). Additionally, various studies suggest a significant association between elevated TGF-β gene expression and autoimmune disease activity, underscoring its involvement in disease progression (Zaninoni et al., 2023). Currently, four primary genetic polymorphisms of TGF-β1 have been identified: 800G > A, −509C > T, +869T > C, and +915G/C. Among these, the TGF-β1 T869C polymorphism is the most extensively studied polymorphism in the TGF-β1 gene. Numerous studies have demonstrated an association between the T869C polymorphism and susceptibility to various autoimmune diseases such as rheumatoid arthritis, systemic lupus erythematosus, and systemic sclerosis. This polymorphism, located in the first exon of TGF-β1, involves an amino acid substitution from leucine (Leu) to proline (Pro), which may affect the function of the TGF-β1 signal peptide and its immune-modulating effects (Sugiura et al., 2002). The functional significance of this polymorphism in the regulation of TGF-β1 activity has been a central focus of research. Epidemiological evidence further indicates a strong correlation between the T869C polymorphism and the incidence of autoimmune diseases in Asian populations, underscoring the relevance of examining this polymorphism within this demographic. The objective of this study was to undertake a more comprehensive evaluation of the link between TGF-β1 T869C polymorphism and susceptibility to autoimmune diseases by encompassing a wider spectrum of autoimmune conditions and incorporating the latest literature. Investigating the T869C polymorphism not only facilitates comparison and analysis with previous research findings but may also uncover novel therapeutic targets and deepen our understanding of disease mechanisms. Consequently, based on the extensive research in the literature, biological importance, genetic significance, epidemiological data, consistency in study design, clinical relevance, innovation, the necessity of the study, and statistical power, this meta-analysis selected the T869C polymorphism as the focus of this research to explore the role of the TGF-β1 T869C genetic polymorphism in the development of autoimmune diseases, providing a basis for the development of diagnostics and treatments for autoimmune diseases.

Research findings suggest that in Asian populations, carriage of the T allele may confer a risk factor for autoimmune diseases, with individuals possessing this allele being more likely to develop rheumatoid arthritis (RA). Following the initial exploration by Alayli et al. on the association between the TGF-β1 T869C polymorphism and RA in a Turkish population, this polymorphism has garnered increasing attention in the context of autoimmune diseases, providing early evidence for a link between the TGF-β1 T869C polymorphism and an increased risk of RA, which laid the groundwork for subsequent studies (Alayli et al., 2009). Mattey et al. demonstrated that the TGF-β1 T869C polymorphism correlates with disease outcomes and mortality rates among patients with RA, underscoring the potential impact of the T allele on RA severity (Mattey et al., 2005). In the 2010s, Zhang L and colleagues published a meta-analysis encompassing 21 studies, identifying a potential association between the TGF-β1 T869C promoter polymorphism and RA, particularly within Asian populations (OR = 0.81, P = 0.003) (Zhang et al., 2013). This analysis provides compelling evidence supporting the connection between the TGF-β1 T869C polymorphism and increased autoimmune disease risk in Asians, corroborating previous findings and elucidating TGF-β1’s pivotal role in immune response regulation and inflammation promotion. However, the study was not without its limitations, including the inclusion of studies that failed to exclude cases that did not conform to the Hardy-Weinberg equilibrium (HWE), indicating a need for further investigation. Building on this, Hussein et al. highlighted the possible link between the TGF-β1 T869C polymorphism and RA disease progression, whereas Saad et al. presented genetic evidence of associations between multiple polymorphisms, including TGF-β1 and RA. Shaker et al. reaffirmed the link between the TGF-β1 T869C polymorphism and RA susceptibility, with findings supported across a broad spectrum of Asian populations. Recently, Zhu et al. investigated the TGF-β1 mechanism in RA and revealed that TGF-β1 facilitates the migration and invasion of fibroblast-like synoviocytes via the TGF-β1/Smad signaling pathway (Zhu et al. 2019). Their results align with those of this meta-analysis, indicating that the T allele is a risk factor for RA in Asian populations. Furthermore, the T allele of TGF-β1 may be correlated with inflammatory activity, nodular disease, and adverse prognosis in patients with RA. Hassan et al. observed a link between the TGF-β1 T869C polymorphism and disease activity in Egyptian patients with RA, reinforcing the role of the T allele in RA pathogenesis. Collectively, these studies suggest that the TGF-β1 T869C polymorphism could serve as a predictive biomarker for RA and other autoimmune diseases, which is crucial for the early diagnosis and personalized treatment strategy development. While existing research has offered valuable insights, further investigation is essential to explore the interactions between the TGF-β1 T869C polymorphism and additional genetic and environmental factors as well as their impact on autoimmune disease development and progression.

This meta-analysis included 32 case–control studies, including 4,304 cases and 4,664 controls, as documented in 31 articles. The objective of this study was to evaluate the correlation between the TGF-β1 T869C polymorphism and the propensity for autoimmune diseases. Both allele and homozygous models demonstrated significant associations with susceptibility to autoimmune diseases. An ethnic subgroup analysis revealed that across all genetic models, the TGF-β1 T869C polymorphism was markedly associated with autoimmune disease susceptibility in Asian populations, a finding not observed in Caucasian or mixed-race populations. To ascertain the reliability of the study, the researchers evaluated publication bias using both Egger’s and Begg’s tests in addition to performing a sensitivity analysis.

This meta-analysis had several limitations. It included only six studies on systemic lupus erythematosus, seven on systemic sclerosis, one on Sjögren’s syndrome, and a single study on juvenile idiopathic arthritis. The limited number of studies and their inherent heterogeneity necessitate further verification to substantiate the correlation between TGF-β1 T869C polymorphism and autoimmune disease susceptibility. Autoimmune diseases are influenced by a multitude of factors, including genetics, sex, and environmental factors. Additionally, the absence of access to the original data in this study restricts the capacity for a more comprehensive analysis of the interactions between the TGF-β1 T869C polymorphism and environmental influences, lifestyle, and clinical manifestations. No predictive models have been established to account for potential confounding factors, such as sex, age, and environmental conditions. Future research should focus on gene-gene and gene-environment interactions to more effectively assess the relationship between the TGF-β1 T869C polymorphism and autoimmune disease susceptibility. Moreover, the study did not incorporate TGF-β1 levels in its data analysis, which precludes the elucidation of the relationship between TGF-β1 level variations and autoimmune diseases. While this study does not directly evaluate TGF-β1 levels, the existing literature indicates that fluctuations in TGF-β1 levels are associated with disease activity in rheumatoid arthritis (RA). TGF-β1, which plays a dual role in regulating immune responses and suppressing inflammation, may be associated with RA severity and progression. Asian populations, with their distinct genetic backgrounds and environmental exposures, may exhibit a unique relationship between TGF-β1 gene polymorphisms and RA susceptibility. Environmental factors, including infections, lifestyle, and dietary habits, may interact with genetic factors to collectively influence the expression and function of TGF-β1. This study underscores the importance of measuring TGF-β1 levels in future research, particularly in Asian patients with RA. Gaining insight into the relationship between TGF-β1 levels and the T allele could aid in uncovering the molecular mechanisms of RA and inform the development of novel treatment strategies. However, due to the constraints of this study’s design and the data available, direct measurement data of TGF-β1 levels were not provided. This study primarily relies on genetic data, offering insights into the relationship between TGF-β1 gene polymorphisms and RA susceptibility.

In summary, despite certain limitations, the discovery of a significant association between the T allele of the TGF-β1 gene and susceptibility to disease in Asian patients with rheumatoid arthritis (RA) is a noteworthy finding that merits further investigation. This meta-analysis, examining the link between the TGF-β1 T869C polymorphism and autoimmune disease susceptibility across 32 studies (31 articles), substantially expands the scope of previous meta-analyses, thereby bolstering the statistical strength of the collective analysis. Within this dataset, 17 studies specifically addressed rheumatoid arthritis, offering more robust evidence to affirm the connection between the TGF-β1 T869C polymorphism and RA susceptibility. Consequently, the meta-analysis concluded that individuals carrying the T allele might be at an elevated risk of developing autoimmune diseases, particularly RA, in Asian populations. The heightened risk of RA development among patients with the T allele underscores the need for additional research on this genetic correlation.

## Data Availability

The original contributions presented in the study are included in the article/Supplementary Material, further inquiries can be directed to the corresponding author.
