# Supplementary material for: Comprehensive systematic review and meta-analysis of the TGF-β1 T869C gene polymorphism and autoimmune disease susceptibility
Source: Front Genet. 2025 Feb 20;16:1502921. doi: 10.3389/fgene.2025.1502921 (PMC11882589; doi:10.3389/fgene.2025.1502921)
Supplement: Supplementary file 1 [file Table1.docx]

Supplementary Material

# Supplementary Data

#

**Table 1** Summary plot of recessive gene models (TT over TC+CC)

#

**Table 2** Summary plot of dominant gene models (TT+ TC over CC)

#

**Table 3** Summary plot of allele gene models (T over C)

**Table 4** Summary plot of homozygous gene models (TT over CC)

**Table 4** Summary plot of dominant gene models (TC over CC)

# 2 Supplementary Figures and Tables

**
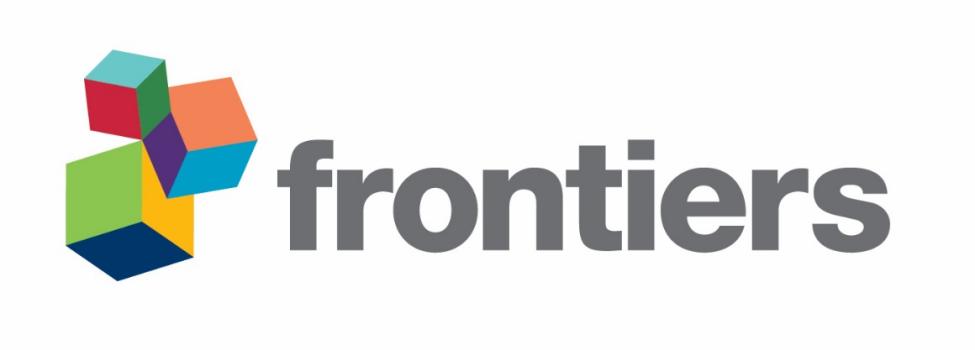
**

**Figures**





**Figure 1** Schematic diagram of the literature screening process


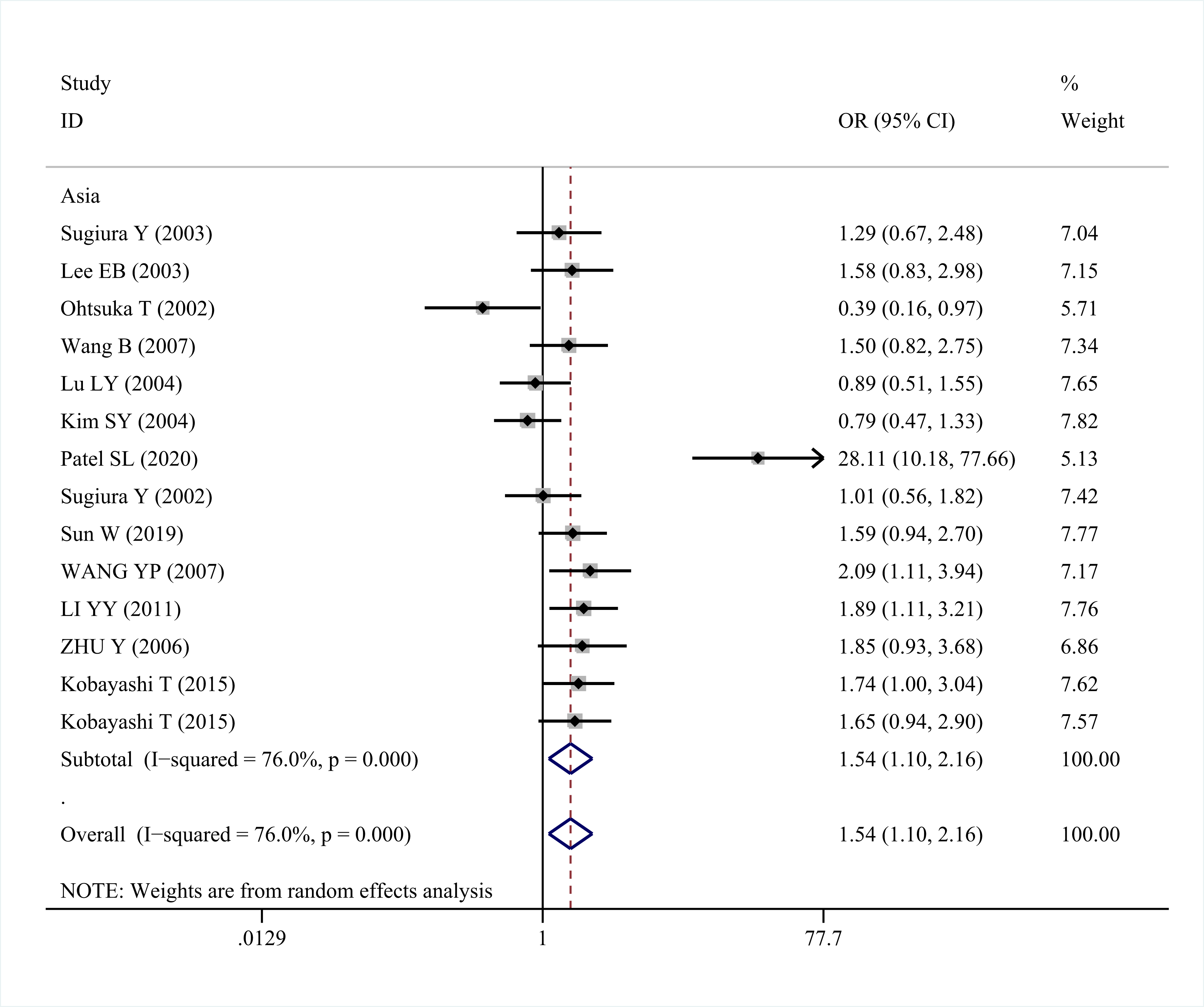


**Figure 2** Forest map of Asian recessive gene model


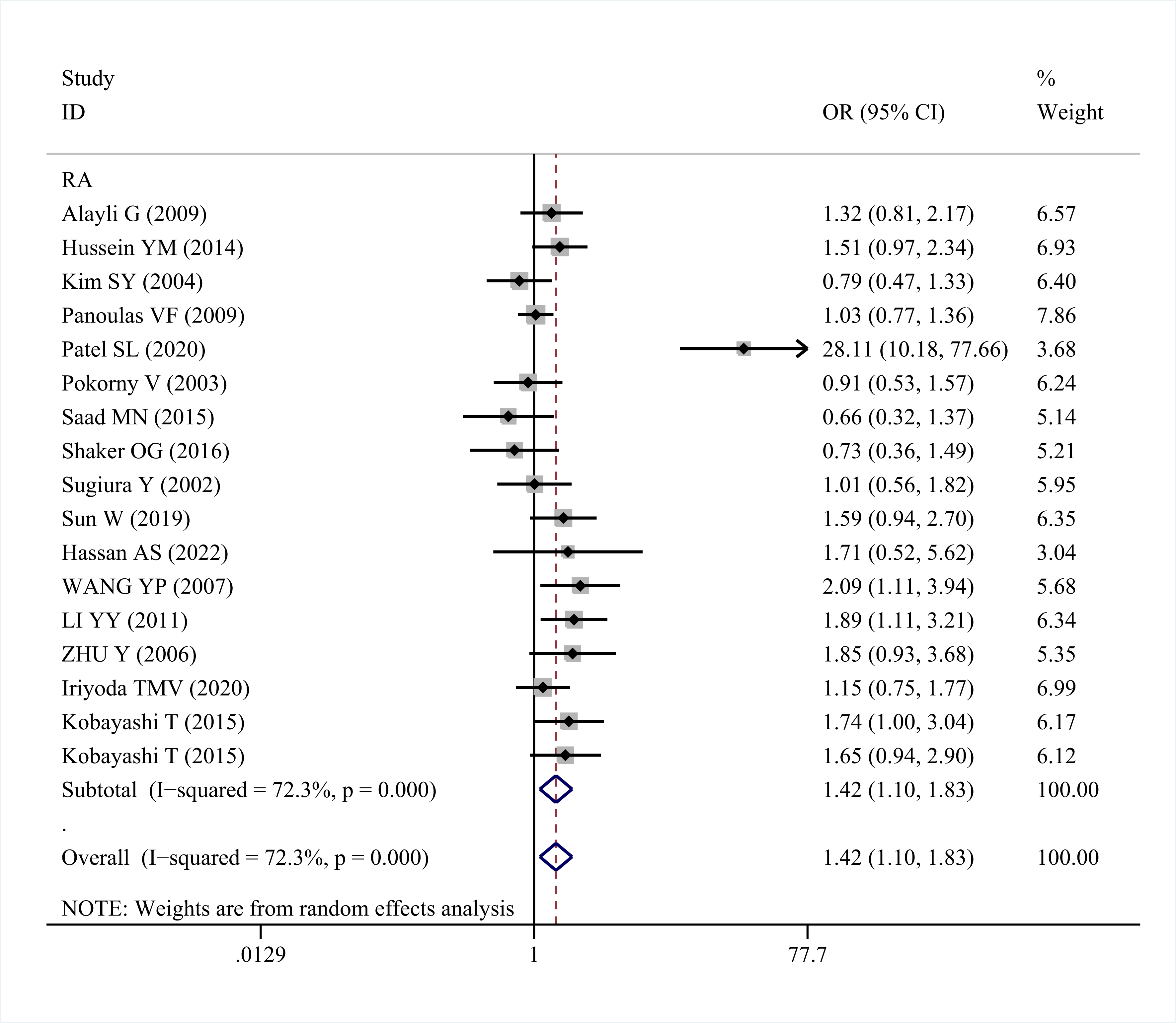


**Figure 3** Forest map of RA recessive gene model


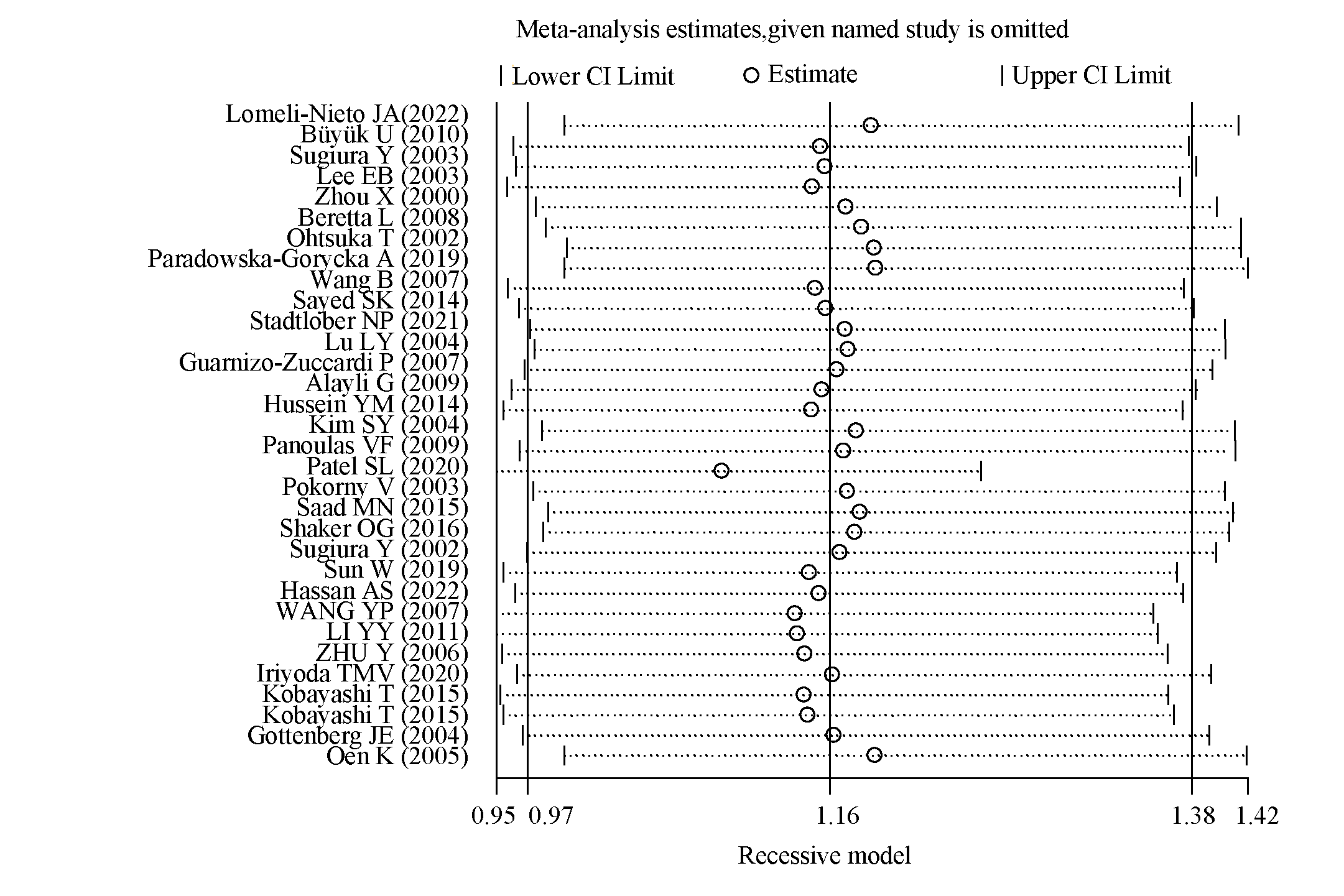


**Figure 4** Sensitivity analysis of TGF-β1 T869C polymorphism


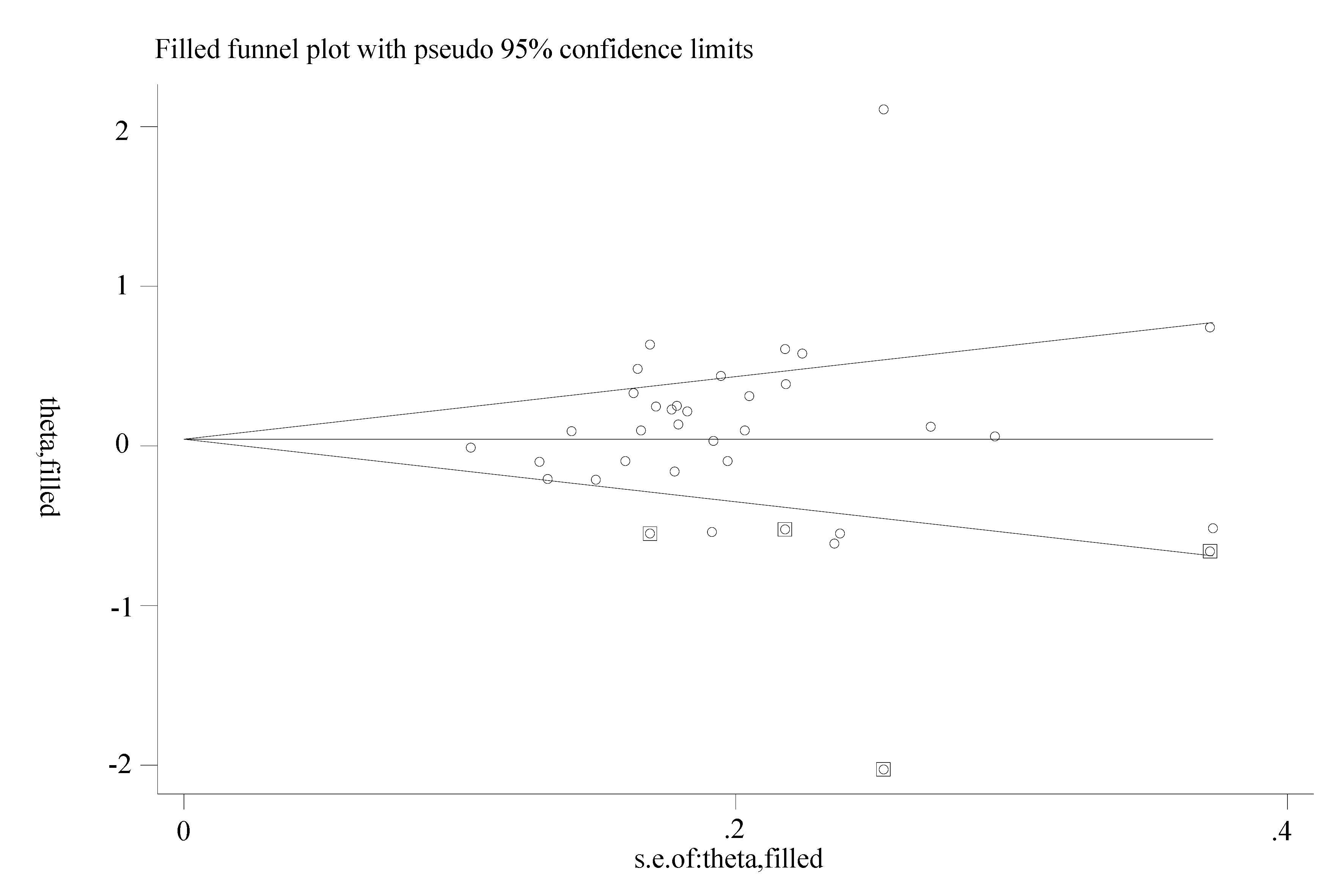


**Figure 5** Begg funnel plot
